# Personalized medicine for pathological circadian dysfunctions

**DOI:** 10.3389/fphar.2015.00125

**Published:** 2015-06-19

**Authors:** Rachel L. Skelton, Jon M. Kornhauser, Barbara A. Tate

**Affiliations:** ^1^Insight Genetics, Inc.Nashville, TN, USA; ^2^PhosphoSite, Cell Signaling Technology, Inc.Danvers, MA, USA; ^3^Armada Therapeutics, Inc.Brooklyn, CT, USA

**Keywords:** personalized medicine, circadian disorders, circadian therapeutics, patient enrollment, companion diagnostics, targeted therapeutics

## Abstract

The recent approval of a therapeutic for a circadian disorder has increased interest in developing additional medicines for disorders characterized by circadian disruption. However, previous experience demonstrates that drug development for central nervous system (CNS) disorders has a high failure rate. Personalized medicine, or the approach to identifying the right treatment for the right patient, has recently become the standard for drug development in the oncology field. In addition to utilizing Companion Diagnostics (CDx) that identify specific genetic biomarkers to prescribe certain targeted therapies, patient profiling is regularly used to enrich for a responsive patient population during clinical trials, resulting in fewer patients required for statistical significance and a higher rate of success for demonstrating efficacy and hence receiving approval for the drug. This personalized medicine approach may be one mechanism that could reduce the high clinical trial failure rate in the development of CNS drugs. This review will discuss current circadian trials, the history of personalized medicine in oncology, lessons learned from a recently approved circadian therapeutic, and how personalized medicine can be tailored for use in future clinical trials for circadian disorders to ultimately lead to the approval of more therapeutics for patients suffering from circadian abnormalities.

## Introduction

The approval of HETLIOZ® (tasimelteon) for Non-24 disorder has reignited an interest in developing therapeutics for circadian abnormalities. However, as the history of tasimelteon demonstrates, the path to approval of a drug for a circadian dysfunction is not an easy one. One component of the challenge is the ability to match the therapeutic approach to the patient most likely to benefit from the intervention, a model often called personalized medicine. Patient selection or optimization of therapy is guided in some indications by genetic or biochemical markers which point to the underlying molecular mechanisms causing disease. In central nervous system (CNS) disorders, genetic markers have been difficult to pinpoint, possibly because the clinical symptoms actually represent multiple diseases with different molecular drivers. However, treating circadian dysfunction has an advantage over other CNS disorders, in that the phenotype of the disorder is relatively easy to measure, is highly translatable from animal models to humans, and modulation of the phenotype can be quantified, providing an accessible proof of mechanism biomarker. Here we review the recent history of clinical trials for circadian disorders; conduct a comparison to oncology, where personalized approaches have greatly improved success for therapeutics; and discuss the challenges that remain in circadian medicine to incorporate personalized approaches to improve the approval rate of medicines for circadian disorders.

## Background

Circadian clocks regulate a plethora of biological processes. Cellular processes such as gene expression and physiological processes such as body temperature, hormone and neurotransmitter release, as well as behaviors including activity, sleep, learning and memory are under circadian control. Recent advances in understanding the molecular components of the clock, together with global transcriptome approaches, provided insight into the vast extent of the genetic landscape regulated by the clock, helping to explain at the molecular level how such a variety of physiological systems are circadian-regulated. The cycling clock proteins (BMAL1, CLOCK, PER-1 and -2, CRY-1 and -2) directly bind to gene regulatory elements and regulate thousands of transcripts in multiple tissues in a temporally coordinated manner (Koike et al., 2012). Recent genome-wide studies have shown that an astonishing 43% of genes are transcribed with a rhythmic pattern in a largely tissue-specific manner (Zhang et al., 2014). Finally, epigenetic mechanisms of gene regulation are also clock-controlled; a large number of non-coding RNAs are rhythmically expressed. Among these cycling mRNAs, only about half are regulated by *de novo* transcription; post-transcriptional regulation is also modulated by circadian input.

This recent understanding of the enormous impact of the clock on the human organism and the elucidation of the molecular components of the clock provide the pharmaceutical industry with potential targets that should prove fruitful for the discovery and development of new therapeutics. As of late 2014, there were 34 open clinical trials listed at https://clinicaltrials.gov/ that included the keyword “circadian.” The purpose of several of these trials is to phenotype circadian rhythms in specific patient populations, for example the NIAAA study on the circadian dopamine rhythm in cocaine addicts (Table 1, NCT02233829) and the UCSF study on sleep disruption and delirium (Table 1, NCT01280097). Other trials are studying behavioral interventions to alter circadian rhythms, for example a study at Rhode Island Hospital using chronobiological interventions to treat post-partum depression (Table 1, NCT02053649). Several studies are testing the efficacy of melatonin in various patient populations or the efficacy of the approved drugs Modafinil, Circadin, and tasimelteon for new indications. Disappointingly, no trials listed novel agents for primary circadian disorders, such as advanced- or delayed-phase syndromes, or for circadian disorders secondary to other diseases, such as sundowning in Alzheimer's disease patients, despite the prevalence of these disorders. Sundowning, for instance, has been reported in 2.4–25% patients diagnosed with Alzheimer's disease (AD). Thus, it appears that drug development for circadian dysfunction remains an under-invested area, although the growing understanding of the profound impact of circadian patterns of expression of the majority of human genes in both healthy and sick individuals may stimulate research into potential therapies for circadian disorders. Several major pharmaceutical companies appear to be invested in programs aimed at molecular targets associated with circadian biology. These including programs on inhibitors of casein kinase 1 (CK1, Pfizer, and Amgen) (Sprouse et al., 2010; Long et al., 2012), which is also a target of interest in oncology. In addition, the growing appreciation of the intimate role of clock proteins in metabolism is reflected in research programs in major pharmaceutical and several biotech companies, including projects examining Cry1 [Griebel et al., 2014; Reset Therapeutics (http://resettherapeutics.com/programs/)] and REV-ERB (Kojetin and Burris, 2014).

**Table 1:** **Open drug intervention clinical trials in clinicaltrials.gov that include “circadian,” “clock,” or “chronobiology” as a keyword**.

**Sponsor/Collaborators**	**Interventions**	**Conditions**	**Phase**	**NCT Number**
University of Chicago	Drug: exenatide and Placebo	Type 2 diabetes; sleep disordered breathing	NP	NCT01136798
Xinhua Hospital; Shanghai Jiao Tong University School of Medicine	Drug: etomidate; midazolam; propofol	Congenital hydronephrosis; congenital choledochal cyst; fracture	Phase 4	NCT02013986
Universitätsklinikum Hamburg-Eppendorf	Drug: Melatonin 2 mg and Placebo	Healthy night shift workers, sleep disorders	Phase 3	NCT02108353
Mount Sinai School of Medicine; National Institute of Mental Health (NIMH)	Drug: Modafinil and Placebo	Bipolar disorder	Phase 4	NCT01965925
Stanford University; Patient Centered Outcome Research Institute	Other: CONV care for the diagnosis and treatment of sleep disorders Other: PCCM for the diagnosis and treatment of sleep disorders	Obstructive sleep apnea of adult; insomnia; circadian rhythm sleep disorder, unspecified type; restless legs syndrome; narcolepsy and hypersomnia	NP	NCT02037438
National Institute on Alcohol Abuse and Alcoholism (NIAAA); National Institutes of Health Clinical Center (CC)	Drug: brain dopamine reactivity methylphenidate; brain dopamine receptor C-11 raclopride	Cocaine abuse	Phase 0	NCT02233829
Rhode Island Hospital; The Depressive and Bipolar Disorder Alternative Treatment Foundation	Behavioral: triple chronotherapy; usual care	Depression; major depressive disorder; post-partum depression	NP	NCT02053649
Hopital Foch	Drug: propofol; remifentanil	Anesthesia, general	Phase 3	NCT00896714
University of California, San Francisco; Masimo Labs	NP	Delirium; sleep disorders, circadian rhythm	NP	NCT01280097
National Human Genome Research Institute (NHGRI); National Institutes of Health Clinical Center (CC)	Drug: dTR Melatonin (NIH CC PDS); melatonin CR Device: phototherapy (bright light)	Developmental delay disorders; chromosome deletion; mental retardation; sleep disorders, circadian rhythm; self-injurious behavior	Phase 1	NCT00506259
Oregon Health and Science University	Drug: melatonin Behavioral: regular sleep schedule; light	Insomnia; blindness; daytime sleepiness	NP	NCT00911053
Hospital de Clinicas de Porto Alegre	Drug: melatonin and Placebo; amitriptyline and Placebo; melatonin and amitriptylin	Fibromyalgia	Phase 2 Phase 3	NCT02041455
Paracelsus Medical University; Technische Universität München	Drug: testosterone supplementation	Circadian; exercise; testosterone	NP	NCT02134470
Sogo Rinsho Médéfi Co., Ltd.; Takeda	Drug: azilsartan; amlodipine	Hypertension	NP	NCT01762501
Ann & Robert H Lurie Children's Hospital of Chicago; Children's Research Institute	Drug: prednisone and Placebo	Duchenne Muscular Dystrophy (DMD)	Phase 2	NCT02036463
Brigham and Women's Hospital	Biological: melatonin and Placebo	Delayed sleep phase disorder; jet-lag; shift-work disorder	NP	NCT00950885
Oregon Health and Science University; Eunice Kennedy Shriver National Institute of Child Health and Human Development (NICHD)	Dietary supplement: melatonin Biological: melatonin	Blindness	NP	NCT00691444
Prince of Songkla University	Device: selective laser trabeculoplasty Drug: travoprost	Intraocular pressure	Phase 4	NCT02105311
Neurim Pharmaceuticals Ltd.	Drug: circadin 2/5/10 mg and Placebo	Sleep disorders	Phase 3	NCT01906866
Hospices Civils de Lyon	Drug: melatonin and Placebo	Sleep disorders	Phase 2	NCT01993251
University of Vigo	Drug: aspirin	Type 2 diabetes	Phase 4	NCT00725127
University of Bergen	Drug: paracetamol and buprenorphine; paracetamol and Placebo; buprenorphine and Placebo	Depression; pain; dementia	Phase 4	NCT02267057
University of Pittsburgh; National Heart, Lung, and Blood Institute (NHLBI)	Behavioral: modified ME intervention; education only	Sleep apnea, obstructive	Phase 1	NCT01377584
Herlev Hospital	Drug: melatonin, N-acetyl-5-methoxytryptamine; isotonic saline, natrium chloride	Acute myocardial infarction; ischemia-reperfusion injury	Phase 2	NCT01172171
Vanda Pharmaceuticals	Drug: tasimelteon	Smith-Magenis syndrome; circadian	Phase 2	NCT02231008
University of British Columbia	Drug: melatonin and Placebo	Delirium	Phase 4	NCT02282241
University of Michigan; University of Pennsylvania; Washington University Early Recognition Center	Drug: ISOFLURANE- experimental arm Other: control group: cognitive testing	Post-operative cognitive dysfunction	NP	NCT01911195
Haukeland University Hospital	Drug: Solu-Cortef; Cortef	Addison disease	Phase 1 Phase 2	NCT02096510
Charite University; Technische Universität München; University of Erlangen-Nürnberg Medical School; Praxis für Neurologie und Psychiatrie am Prinzregentenplatz, München; Technische Universität Berlin	Behavioral: patient centered structured support program	Mini-stroke	NP	NCT01586702
Teva Pharmaceutical Industries; United BioSource Corporation	Drug: modafinil; armodafinil	Narcolepsy; obstructive sleep apnea; shift work sleep disorder	NP	NCT01792583
Vanda Pharmaceuticals	Drug: tasimelteon	Non-24-H sleep-wake disorder	Phase 3	NCT01429116
Vanda Pharmaceuticals	Drug: tasimelteon	Non 24 H sleep wake disorder	Phase 3	NCT01218789
Aretaieion University Hospital; Baxter Healthcare Corporation	Procedure: maintenance with desflurane Procedure: maintenance with propofol	Anesthesia; surgery; sleep disorders	NP	NCT02061514
Endo Pharmaceuticals	Drug: morphine Sulfate 30 mg; Oxycodone 20 mg; Morphine 45 mg; Oxycodone 30 mg; Morphine sulfate 15 mg; Oxycodone 10 mg; morphine Sulfate 30 mg; Oxycodone 15 mg	Chronic around the clock opioid users	Phase 2	NCT01871285
Mundipharma Research GmbH & Co KG	Drug: Oxycodone/Naloxone prolonged release (OXN PR) tablets; oxycodone prolonged release (OxyPR) tablets	Pain|Constipation	Phase 3	NCT01438567
Brigham and Women's Hospital|National Center for Complementary and Integrative Health (NCCIH)	Drug: vitamin B12	Sleep disorders, circadian rhythm		NCT00120484
Endo Pharmaceuticals|BioDelivery Sciences International	Drug: EN3409	Low back pain|Osteoarthritis|Neuropathic pain	Phase 3	NCT01755546
Mundipharma Research GmbH & Co KG	Drug: Oxycodone; Naloxone	Malignant pain|Non-malignant pain	Phase 2|Phase 3	NCT02321397
Purdue Pharma LP	Drug: Oxycodone/Naloxone controlled-release; Placebo	Low back pain	Phase 3	NCT01358526
Janssen Pharmaceutical K.K.	Drug: tapentadol ER; morphine SR	Neoplasms	Phase 3	NCT01309386
Purdue Pharma LP	Drug: Oxycodone/Naloxone controlled-release; Oxycodone HCl controlled-release; Placebo	Low back pain	Phase 3	NCT01427270
Purdue Pharma LP	Drug: oxycodone/naloxone controlled-release; oxycodone HCl controlled-release; Placebo	Low back pain	Phase 3	NCT01427283
Oregon Health and Science University|Forest Laboratories	Drug: Placebo/escitalopram	Depression		NCT01214044
Technische Universität München|Cephalon	Drug: modafinil (Vigil); Placebo	Depression	Phase 2	NCT00670813
University of Copenhagen| Rigshospitalet, Denmark	Drug: erythropoietin (Epoetin-beta, NeoRecormon); erythropoietin (Epoetin-beta, NeoRecormon); Placebo	Renal effects	Phase 1	NCT01584921
Norwegian University of Science and Technology|St. Olavs Hospital|Fondazione IRCCS Istituto Nazionale dei Tumori, Milano|L'Hospitalet de Llobregat|University Hospital, Bonn|Cantonal Hospital of St. Gallen|Maastricht University Medical Center|Flinders University	Drug: intranasal fentanyl spray; slow release morphine	Cancer|Pain	Phase 3	NCT01906073
Endo Pharmaceuticals	Drug: oxymorphone IR	Chronic pain	Phase 3	NCT01206907
Mundipharma Research GmbH & Co KG	Drug: laxative	Opioid induced constipation	Phase 4	NCT01957046
Instituto Nacional de Ciencias Medicas y Nutricion Salvador Zubiran	Drug: haloperidol; Placebo; non-pharmacologic measures	Hypoactive delirium	Phase 3	NCT02345902
University of Oklahoma	Drug: memory XL; Placebo	Mild cognitive impairment	Phase 2	NCT00903695
University of Illinois at Chicago|Genentech, Inc.	Drug: ranibizumab (lucentis)	Glaucoma|New onset Glaucoma|Neovascular Glaucoma|New onset neovascular glaucoma	Phase 1|Phase 2	NCT00727038
Universitätsklinikum Hamburg-Eppendorf	Drug: melatonin 2 mg; Placebo	Healthy night shift workers, sleep disorders	Phase 3	NCT02108353
Eunice Kennedy Shriver National Institute of Child Health and Human Development (NICHD)|National Institutes of Health Clinical Center (CC)	Drug: hydrocortisone; Placebo; hydrocortisone and melatonin; melatonin	Jet lag syndrome	Phase 2	NCT00097474
University of California, San Diego|California Breast Cancer Research Program	Device: light box (litebook)	Breast cancer		NCT00478257
St. Olavs Hospital	Drug: fentanyl	Chronic pain|Cancer	Phase 1|Phase 2	NCT01248611
National Heart, Lung, and Blood Institute (NHLBI)	Drug: melatonin; methylxanthine Device: light therapy	Sleep disorders, circadian rhythm		NCT00387179
Takeda	Drug: ramelteon; Placebo	Circadian dysregulation	Phase 4	NCT00492011
AstraZeneca	Drug: AZD1386; Placebo	Pain|Esophageal sensitivity	Phase 1	NCT00711048
Delray Medical Center	Drug: IV Ibuprofen	Pain	Phase 4	NCT02152163
Attikon Hospital	Drug: sugammadex; neostigmine/atropine	Post-operative cognitive dysfunction		NCT02419352
Mundipharma Research GmbH & Co KG	Drug: OXN PR followed by OxyPR tablets; OxyPR followed by OXN PR tablets	Severe chronic pain	Phase 2	NCT01915147
Greater Houston Retina Research	Drug: ranibizumab (lucentis)	Ischemic central retinal vein occlusion	Phase 1	NCT00406471
Pfizer	Drug: donepezil	Dementia, vascular|Dementia, mixed	Phase 3	NCT00174382
Pfizer	Drug: morphine sulfate extended release capsules	Pain	Phase 4	NCT00640042
Meander Medical Center|Dutch Kidney Foundation	Drug: melatonin tablet 3 mg once daily; Placebo comparator	Sleep Problems|Haemodialysis	Phase 3	NCT00388661
Meander Medical Center|Dutch Kidney Foundation	Drug: melatonin	Hemodialysis|Peritoneal dialysis|Sleep problems	Phase 3	NCT00404456
Ever Neuro Pharma GmbH|acromion GmbH|Geny Research Corp.	Drug: cerebrolysin; 0.9% saline solution	Vascular dementia	Phase 4	NCT00947531
Neovii Biotech	Drug: catumaxomab; prednisolone	Cancer|Neoplasms|Carcinoma|Malignant ascites	Phase 3	NCT00822809
Singapore General Hospital|Novartis|National Neuroscience Institute	Drug: exelon (rivastigmine); placebo	Cognitive impairment	Phase 4	NCT00669344
University of Toledo Health Science Campus	Drug: continuous release dopamine agonists	Parkinson disease	Phase 3	NCT00465452
Novartis	Drug: rivastigmine capsule; rivastigmine transdermal patch	Parkinson's disease dementia	Phase 3	NCT00623103
University of Rochester|Forest Laboratories	Drug: namenda	Delirium|Post-operative states	Phase 4	NCT00303433
INSYS Therapeutics Inc	Drug: fentanyl sublingual spray	Cancer|Pain	Phase 3	NCT00538863
Endo Pharmaceuticals|BioDelivery Sciences International	Drug: EN3409; Placebo	Low back pain	Phase 3	NCT01633944
Endo Pharmaceuticals|BioDelivery Sciences International	Drug: EN3409	Low back pain	Phase 3	NCT01675167
Memorial Sloan Kettering Cancer Center	Drug: d-Methadone; Placebo	Pain|Bladder Cancer|Breast Cancer|CNS Cancer|Colon Cancer|Esophageal Cancer|Pancreatic Cancer|Prostate Cancer|Uterine Cancer|Head and neck Cancer|Eye Cancer|Otorhinolaryngologic neoplasms	Phase 1|Phase 2	NCT00588640
Neurim Pharmaceuticals Ltd.	Drug: melatonin (circadin); Placebo	Non-24 H sleep-wake disorder|Blindness	Phase 2	NCT00972075
National Eye Institute (NEI)	Drug: melatonin	Blindness		NCT00686907
Collegium Pharmaceutical, Inc.	Drug: oxycodone DETERx; Placebo	Chronic low back pain	Phase 3	NCT01685684
Cephalon|Teva Pharmaceutical Industries	Drug: ACTIQ (Oral Transmucosal Fentanyl Citrate [OTFC])	Pain|Cancer|Sickle cell Anemia|Severe burns	Phase 2	NCT00236093
Shaare Zedek Medical Center	Drug: extended-release tramadol; paracetamol	Post-operative pain		NCT01024348
Cephalon|Teva Pharmaceutical Industries	Drug: ACTIQ	Cancer|Breakthrough pain	Phase 2	NCT00236041
Accera, Inc.	Drug: AC-1204; Placebo	Alzheimer's disease	Phase 2|Phase 3	NCT01741194
Mundipharma Pharmaceuticals B.V.	Drug: oxycodone hydrochloride and naloxone hydrochloride combination, prolonged release	Pain	Phase 3	NCT01167127
Melissa Voigt Hansen|University of Copenhagen|Rigshospitalet, Denmark|Pharma Nord|Herlev Hospital	Drug: melatonin (N-acetyl-5-methoxytryptamine); Placebo	Breast cancer|Depression	Phase 2|Phase 3	NCT01355523
Mundipharma Pharmaceuticals B.V.	Drug: oxycodone and naloxone	Pain	Phase 3	NCT01167699
James Graham Brown Cancer Center|University of Louisville	Drug: Fentanyl Citrate Nasal Spray (FCNS)	Pain	Phase 4	NCT01839552
Kaplan Medical Center	Drug: oxycodone 10 mg	Elective laproscopic bilateral inguinal Hernia|Elective laproscopic cholecystectomy	Phase 4	NCT00480142
Brigham and Women's Hospital|Takeda	Drug: ramelteon; Placebo	Healthy		NCT00595075
Loma Linda University	Drug: morphine PCA started at the end of surgery, 1 Percocet 1/325 mg every 4 h; may receive a second Percocet if needed. For the 30 ml ropivacaine the intervention would be the subject can request extra pain medication which would be Percocet and/or morphine PCA	Post-op pain		NCT01939379
Vanda Pharmaceuticals	Drug: tasimelteon 20 mg capsule; tasimelteon 2 mg I.V.	Non-24-H-sleep-wake disorder	Phase 4	NCT02130999
Oregon Health and Science University	Drug: melatonin	Insomnia|Blindness|Daytime sleepiness		NCT00911053
Erasmus Medical Center|ZonMw: The Netherlands Organization for Health Research and Development	Drug: enalapril/hydrochlorothiazide; Placebo	Essential hypertension	Phase 4	NCT02214498
National Institute on Alcohol Abuse and Alcoholism (NIAAA)|National Institutes of Health Clinical Center (CC)	Drug: brain dopamine reactivity; brain dopamine receptor	Cocaine abuse	Phase 0	NCT02233829
Boehringer Ingelheim	Drug: telmisartan; ramipril	Hypertension	Phase 4	NCT00274612
Ottawa Heart Institute Research Corporation|Schering-Plow|Medtronic	Drug: eptifibatide facilitated PCI	Myocardial infarction	Phase 3	NCT00251823
Boehringer Ingelheim	Drug: telmisartan combined with hydrochlorothiazide (80/12.5 mg); valsartan combined with hydrochlorothiazide (160/12.5 mg)	Hypertension|Diabetes mellitus, Type 2	Phase 4	NCT00239538
US WorldMeds LLC|National Institute on Drug Abuse (NIDA)	Drug: lofexidine HCl	Renally impaired subjects	Phase 1	NCT02313103
University of Washington|Paul G. Allen Family Foundation	Drug: botox; normal saline	Painful bladder syndrome|Interstitial cystitis	Phase 4	NCT00194610
Children's Hospital of Philadelphia|Bayer|University of Pennsylvania	Drug: paracervical nerve block; sham paracervical block	Pain	Phase 4	NCT02352714
University of Alabama at Birmingham|National Heart, Lung, and Blood Institute (NHLBI)	Drug: losartan	Anemia, sickle cell|Sickle cell disease|Kidney disease|Hypertension| Proteinuria	Phase 2	NCT02373241
University of Alberta|Vancouver Coastal Health Research Institute	Drug: warfarin	Atrial fibrillation|Thrombus due to heart valve prosthesis|Deep venous thrombosis|Thromboembolism|DVT	Phase 4	NCT02376803
National Heart, Lung, and Blood Institute (NHLBI)	Drug: melatonin; methylxanthine| Procedure: light therapy	Sleep disorders, circadian rhythm		NCT00387179
Orphan Medical	Drug: sodium oxybate	Narcolepsy	Phase 3	NCT00049803
National Institute of Mental Health (NIMH)	Drug: low-dose sodium oxybate; high-dose sodium oxybate; low-dose zolpidem; high-dose zolpidem; Placebo	Sleep		NCT00777829
Massachusetts General Hospital	Drug: ramelteon; Placebo	Huntington's disease|Parkinson's disease|Dementia with lewy bodies|Sleep disorders|Circadian dysregulation		NCT00907595
Takeda	Drug: ramelteon; Placebo	Circadian dysregulation	Phase 4	NCT00492011
Massachusetts General Hospital	Drug: zolpidem CR; Placebo	Dementia|Alzheimer disease|Dementia, vascular|Sleep disorders|Circadian dysregulation		NCT00814502
Vanda Pharmaceuticals	Drug: tasimelteon; Placebo	Non-24-H sleep-wake disorder	Phase 3	NCT01163032
Vanda Pharmaceuticals	Drug: tasimelteon; Placebo	Non-24-H sleep-wake disorder	Phase 3	NCT01430754
Cephalon|Teva Pharmaceutical Industries	Drug: PROVIGIL 200 mg; armodafinil 250 mg; armodafinil 200 mg; armodafinil 150 mg; Placebo	Chronic shift work sleep disorder	Phase 3	NCT00236080
Child Psychopharmacology Institute	Drug: risperidone	Sleep disorders, circadian rhythm|Insomnia|psychomotor agitation		NCT00723580
Cephalon|Teva Pharmaceutical Industries	Drug: CEP-10953 (Armodafinil)	Narcolepsy|Sleep apnea, Obstructive|Sleep apnea Syndromes|Shift-work sleep disorder	Phase 3	NCT00078312
Vanda Pharmaceuticals	Drug: tasimelteon	Smith-Magenis syndrome|Circadian	Phase 2	NCT02231008
Vanda Pharmaceuticals	Drug: tasimelteon	Non-24-H sleep-wake disorder	Phase 3	NCT01429116
Takeda	Drug: ramelteon; Placebo	Sleep disorders, circadian rhythm	Phase 2	NCT00593736
Boehringer Ingelheim	Drug: pharmaton caplets; Placebo	Sleep disorders, circadian rhythm	Phase 2	NCT02199847
Sheba Medical Center	Drug: melatonin	Delayed sleep phase syndrome	Phase 1	NCT00282061
Cephalon|Teva Pharmaceutical Industries	Drug: armodafinil 150 mg/day; Placebo	Excessive sleepiness|Shift work sleep disorder	Phase 3	NCT00080288
National Human Genome Research Institute (NHGRI)|National Institutes of Health Clinical Center (CC)	Drug: dTR melatonin (NIH CC PDS); melatonin CR Device: phototherapy (bright light)	Developmental delay disorders|Chromosome deletion|Mental retardation|Sleep disorders, circadian rhythm|Self injurious behavior	Phase 1	NCT00506259
Brigham and Women's Hospital|Sunovion|Massachusetts General Hospital	Drug: eszopiclone; matching Placebo	Shift-work sleep disorder		NCT00900159
Vanda Pharmaceuticals	Drug: tasimelteon 20 mg capsule; tasimelteon 2 mg I.V.	Non-24-H-sleep-wake disorder	Phase 4	NCT02130999
Neurim Pharmaceuticals Ltd.	Drug: melatonin (circadin); Placebo	Non-24 H sleep-wake disorder|blindness	Phase 2	NCT00972075
Eunice Kennedy Shriver National Institute of Child Health and Human Development (NICHD)|National Institutes of Health Clinical Center (CC)	Drug: hydrocortisone; Placebo; hydrocortisone and melatonin; melatonin	Jet lag syndrome	Phase 2	NCT00097474
Teva Pharmaceutical Industries|United BioSource Corporation	Drug: modafinil/armodafinil	Narcolepsy|Obstructive sleep apnea|Shift work sleep disorder		NCT01792583
Vanda Pharmaceuticals	Drug: VEC-162	Circadian rhythm sleep disorders	Phase 2	NCT00490945
Vanda Pharmaceuticals	Drug: tasimelteon	Non 24 H sleep wake disorder	Phase 3	NCT01218789
Cephalon|Teva Pharmaceutical Industries	Drug: armodafinil 100–250 mg/day	Excessive daytime sleepiness|Narcolepsy|Obstructive sleep apnea/hypopnea syndrome|Chronic shift work sleep disorder	Phase 3	NCT00228553
Brigham and Women's Hospital|National Center for Complementary and Integrative Health (NCCIH)	Drug: vitamin B12	Sleep disorders, circadian rhythm		NCT00120484

This review will discuss why trials for circadian disorders risk a high failure rate, what has been learned from the more recent success with the approval of tasimelteon (HETLIOZ®), and what can be done in the future to reduce the risk of clinical trial failures and improve the chances of delivering medications to patients suffering from circadian disorders.

### Human circadian clocks

Humans, in common with most (if not all) living organisms, have biological clocks that organize the timing of physiological events and keep them synchronized with the external 24-h day. This coordination with the 24-h day led to the term “circadian.” The master circadian clock in mammals resides in the suprachiasmatic nucleus (SCN) of the hypothalamus (Meijer and Rietveld, 1989). Rhythms generated by the SCN clock are autonomous (i.e., they do not depend upon environmental cues), but under normal conditions and in healthy individuals the timing of the internal oscillator is synchronized with the external 24-h day by environmental stimuli, principally light. In addition to the master SCN pacemaker, other tissues and organs also possess cellular clocks that are synchronized by signals from the SCN. These peripheral circadian clocks regulate the timing of many metabolic and cellular processes that are specific to the function of the particular cell type and tissue (Mohawk et al., 2012).

The human clock cycles with a period (*tau*) of nearly, but not exactly, 24 h. When an individual is placed into an environment removed from any time cues (free-running conditions), the inherent period of the internal clock is revealed; *tau* has been measured at 24 h 11 ± 16 min (Czeisler et al., 1999). Environmental light normally keeps the internal clock synchronized or entrained to the external day-night cycle. This synchronization is accomplished by an adjustment in the phase of the oscillation, or phase shift, whenever a disparity exists between the internal “time of day” and external lighting conditions. For example, if the internal clock is slow, or delayed, morning light occurring before the internal clock “anticipates” it will advance the phase of the clock; whereas, in a clock running fast, light in the evening will delay the phase of the clock. Light occurring coincident with the internal “daytime” will not shift the clock. The differential effects of light at different times relative to the internal phase can be measured and plotted as a phase-response curve (PRC). The PRCs of humans and other diurnal mammals compared to nocturnal mammals are generally similar—light in the early subjective night lead to a phase delay, while light in the late subjective night lead to an advance, keeping the internal clock synchronized to sunrise and sunset (Johnson, 1990).

While light is the predominant stimulus that entrains the phase of the clock, a variety of other stimuli also affect entrainment. Of particular relevance in terms of human health and disease, inputs including food consumption, exercise, and social interactions can shift clock phase. These non-photic stimuli generally shift the clock when they occur during the circadian inactive phase (i.e., during the night in humans) (Rosenwasser and Dwyer, 2001).

In recent years, the molecular “gears” of the circadian clock have been elucidated. A series of interlocking negative feedback loops involving transcription, translation, and post-translational phosphorylation form the molecular clockwork. At its core, the transcriptional activators BMAL1, CLOCK and NPAS2 activate the *Period* (Per1 and Per2) and *Cryptochrome* (Cry1 and Cry2) genes (Mohawk et al., 2012). PER and CRY transcripts and proteins gradually accumulate during the daytime, associate with one another and translocate into the nucleus during the evening, and interact with the CLOCK/NPAS2:BMAL1 complex to repress their own transcription. The PER and CRY proteins are progressively phosphorylated by a CK1 kinase during the night, targeting them for ubiquitylation and eventual degradation by the proteasome, relieving their transcriptional autorepression and beginning the cycle again. The timing of this feedback loop takes about 24 h to complete. Because CK1 phosphorylation of PER and CRY regulates the timing of degradation of these protein and the link to a specific human circadian phenotype, CK1 is a target under investigation for its therapeutic potential. Other clock components represent potential drug targets, although little is available in the public domain confirming pharmaceutical investment in these targets.

A secondary or modulatory protein loop modifies the core clock loop. REV-ERBα transcription is also activated by the BMAL1/CLOCK and repressed by CRY/PER, resulting in circadian oscillations of REV-ERBα. In turn, REV-ERBα represses BMAL1 and CLOCK transcription. This REV-ERBα/RORα feedback loop modulates the core circadian clock (Bugge et al., 2012). REV-ERBα has also generated interested as a drug target.

### Circadian rhythms and disease

Underlying genetic mutations have been discovered which lead to abnormal circadian rhythms. In this review we refer to these genetic-driven circadian abnormalities as Primary Circadian Disorders. For example, some cases of advanced sleep phase disorder (ASPD) and delayed sleep phase disorder (DSPD), characterized by a circadian phase that is either “fast” or “slow,” respectively, are due to mutant forms of key clock proteins. Familial forms of ASPD arise from mutations in either CK1 delta or in a phosphorylation site in the PER2 protein targeted by CK1. Specific CK1 variants are also associated with DSPD, and 75% of DSPD patients are homozygous for a shorter allele of PER3 that affects phosphorylation by CK1. These human circadian phenotypes are predicted by the spontaneous mutations in CK1 that result in the circadian period mutants found in hamster, *Tau*, and in Drosophila, *double-time*. In general, it appears that increasing CK1 activity leads to a shortened circadian period. Thus, CK1 activity is an important regulator of circadian timing and a potential target for therapeutic intervention. Clearly, it is critical to take into account the circadian phenotype and phase of an individual to predict clinical outcome in trials of drugs that modulate CK1. As in clinical trials for oncology treatments, companion diagnostic (CDx) tests that genotype for specific clock gene mutations would allow selection of the target patient population. Therefore, as in oncology, these genetic disorders provide the opportunity to demonstrate, as a proof of principle, the efficacy of the drug mechanism in a targeted population, with the goal of expanding therapeutics into Secondary Circadian Disorders (see below).

In addition to mutations in core clock genes, genetic variations that affect the timing of the circadian cycle of humans may also exist. For example, polymorphisms in *Clock* are associated with morning vs. evening preference in humans (Katzenberg et al., 1998). A recent paper describes an association of a Per3 polymorphism with bipolar disorder (Karthikeyan et al., 2014). Diagnostics that detect these kinds of genetic markers would provide a mechanism to enrich enrollment in clinical trials with patients most likely to benefit from specific circadian therapies.

Beyond the genetic disorders discussed above that are the direct result of mutations in clock genes or disrupted entrainment of the circadian system, there is a growing appreciation that circadian abnormalities may be a key core symptom of a variety of diseases including metabolic disorders, mood disorders, and dementia. In this review we refer to circadian abnormalities that are closely associated with another disease as Secondary Circadian Disorders. In Secondary Circadian Disorders, circadian disorganization may present both as a symptom of the disease, and as a potential risk factor contributing to disease pathogenesis (Golombek et al., 2013; Smolensky et al., 2014a,b; Zelinski et al., 2014). An instructive example is the occurrence of circadian abnormalities in patients with metabolic disorder. The growing appreciation of the role of clock proteins in metabolism suggests several potential molecular targets for therapeutics aimed at treating obesity. A feedback between the central circadian clock and peripheral oscillators in liver, skeletal muscle and other tissues helps to coordinate the complex processes of food intake, activity, and lipid homeostasis (Feng and Lazar, 2012). Disruption of these highly regulated interacting rhythms by rotational shift work, for example, is a risk factor for developing metabolic syndrome, obesity, and diabetes mellitus (Feng and Lazar, 2012; Bailey et al., 2014; Maury et al., 2014). While not all obese patients may suffer from circadian disruption, phenotyping, and appropriately regulating the sleep-wake cycles of patients in therapeutic trials for obesity may be critical to uncovering the full potential of a drug. Indeed, individualizing the timing of drug administration may be an unappreciated factor to improve efficacy, especially in a population where sleep-wake disruption is overrepresented.

Abnormal circadian rhythms are also common in patients with mood disorders. Bipolar disorder patients have been reported to have unstable and lower amplitude circadian rhythms (McCarthy and Welsh, 2012; Seleem et al., 2014) while those suffering from major depressive disorder appear to be phase delayed. It has been shown that circadian programs of gene expression are distinctly altered in depressive patients (McCarthy and Welsh, 2012; Karatsoreos, 2014). While the long-standing hypothesis has been that circadian disruption may be a causal factor in these disorders, circadian-based treatments have not always shown pronounced efficacy. There is an ongoing debate regarding the efficacy of agomelatine, an approved treatment of major depressive disorder in Europe and Australia although the drug is not approved for this indication in the US (Gahr, 2014). A recent prospective study suggested that treatment responsiveness was related to circadian phenotype. Patients with major depressive disorder that scored as a morning type were more likely to respond to agomelatine treatment than those that scored as an evening type (Corruble et al., 2013). Thus, treatment regimes informed by an individual patient's circadian phenotype and/or administered at a specific circadian phase might enhance the therapeutic benefit for this chronobiotic.

Seasonal affective disorder (SAD) represents a sub-type of mood disorder closely linked to the circadian system. SAD affects individuals who become depressed during the short daylight periods of winter. One leading hypothesis of the cause of SAD suggests it a results from circadian misalignment (Lewy et al., 2007), or difficulty entraining due to the absence of bright light in morning or evening. Therapy with bright light and melatonin is effective for some SAD patients, and is thought to act by advancing or delaying phase to re-synchronize the clock. Especially in this disorder, treatment aligned with the individual patient's circadian phase is likely to improve outcomes.

In dementia patients, especially those suffering from AD, up to 25% experience sundowning, a disturbing syndrome characterized by agitation, worsening cognitive function, pacing and wandering in the evening/night, and daytime sleepiness. A patient's reduced internal distinction between night and day, caused by the low amplitude oscillation of their circadian clock, appears to contribute to sundowning (Khachiyants et al., 2011). Therapeutics that enhance the amplitude of the circadian oscillator could benefit this patient population.

Finally, the recently approved drug, tasimelteon, is prescribed for patients with Non-24-h sleep-wake disorder (Non-24). Non-24 is a consequence of the failure of the circadian system to entrain to the external 24-h day. A majority of Non-24 patients are blind; and up to 70% of totally blind individuals may suffer from Non-24. The absence of light perception leads to the lack of clock entrainment, and as a result the sleep-wake cycle is free-running. Non-24 is the first circadian disorder for which a pharmaceutical intervention, tasimelteon, has been approved. Because Non-24 patients express a range of free-running *tau* (from 23.7 to 25.3 h) (Dressman et al., 2012), consideration of their circadian phenotypes proved to be key to successful clinical trials (see further discussion of the clinical trial, below).

Considering that circadian rhythmicity is ubiquitous and closely intertwined with both normal physiological processes and disease states, any therapeutic approach targeting a disease (either a “circadian” disorder, or one with rhythmic components) must take into account the timing of the intervention relative to the circadian system of the individual. The experience in drug development for oncology demonstrates the value of a personalized medicine approach to discovering and testing therapeutics.

## Definition of personalized medicine

Personalized medicine can be defined as a targeted prevention and treatment regimen that is developed for an individual using data gathered from medical records and diagnostic analysis of “biomarkers,” or specific biological markers that distinguish one individual from another. Most often, biomarkers are genetic; however, biomarkers can also be phenotypic, such as circadian subtypes (e.g., advance phase or delayed phase syndrome). Ultimately, the goal of personalized medicine is to give the right patient the right treatment at the right time.

Following the completion of the Human Genome Project over 10 years ago, the cost and time for genomic sequencing has decreased exponentially. On average, sequencing of a human genome decreased from $1 billion in 2003 to between $3–5000 today (Personalized Medicine Coalition, 2014), with a time to result from ~7 years decreased to ~2 days. In addition to influencing all of biological research, mapping of the genome has also translated to clinical chemistry, with many therapeutics now designed to target specific proteins, protein classes, or even mutated forms of a protein. In contrast to traditional chemotherapies, which function against any actively dividing cells, targeted therapeutics act on a molecule in the signaling pathway driving a specific tumor (http://www.cancer.gov/cancertopics/factsheet/Therapy/targeted).

The following section will examine the growing field of personalized medicine in oncology, which has focused on targeting the genetic abnormalities driving cancer with targeted therapies. As patients with circadian disorders continue to be profiled both at the phenotypic and molecular levels, the ability to subtype patient populations in order to identify the predicted responsive cohort is critical in ensuring more efficient and more successful circadian clinical trials.

### Personalized medicine in oncology

The oncology field has led to the use of personalized medicine in healthcare, mainly due to the effectiveness of targeted therapeutics specifically developed to inhibit oncogenic driver proteins. Currently, targeted therapeutics are available for patients with melanoma, chronic myeloid leukemia, colon, breast, and lung cancer. In non-small cell lung cancer (NSCLC), mutations in two oncogenic driver genes, EGFR and ALK, make up ~25–40% of all NSCLC cases (Kwak et al., 2010; Melosky, 2014). Two FDA-approved EGFR inhibitors and two FDA-approved ALK inhibitors are commercially available (EGFR: Gilotrif™, Boehringer Ingelheim and Tarceva®, Roche; ALK: Xalkori®, Pfizer and Zykadia™, Novartis). Each of these inhibitors requires testing of patients with a unique FDA-approved diagnostic test (aka CDx) prior to prescription. CDx assays are FDA regulated as Class III Medical Devices (Olsen and Jorgensen, 2014) that were demonstrated during the pivotal trial to show clinical utility in selecting for the responding patient population, were contemporaneously FDA-approved alongside the corresponding therapeutic, and are specified in the drug labeling to be required for use in identifying the target patient population. Therefore, a CDx is the mechanism to identify the right patient for the right drug.

## Personalized medicine growing pains

Use of CDx in the oncology space was initially met with resistance; the thought of narrowing the market to a select patient population does not inherently make financial sense. However, as drugs in development functioned through a more selective mechanism of action, it was clear that identification of responders carrying the genetic target was paramount, as dictated by the FDA. For example, the small molecule Iressa™ (gefitinib, AstraZeneca) was initially approved in Japan in July 2002 and in May 2003 through the FDA Accelerated Approval path for NSCLC. Under the Accelerated Approval guidelines, approved therapeutics are required to complete a post-approval Phase III study. Completion of the obligatory Phase III Iressa Survival Evaluation in Lung Cancer (ISEL) trial in 2004 revealed no improvement in overall survival in patients taking Iressa vs. placebo (Thatcher et al., 2005). However, an academic study published that same year suggested that the subset of Iressa responders correlated to mutations in the tyrosine kinase Epidermal Growth Factor Receptor (EGFR) gene. Retrospective analysis of the ISEL data with genetic testing for mutations in EGFR clearly indicated that patients with mutations in EGFR responded significantly better to Iressa than patients without EGFR mutations; interestingly, the patient cohorts carrying the EGFR mutations were predominantly Asian women with no smoking history (Lynch et al., 2004). In 2005 FDA responded to the Iressa-EGFR findings by allowing for use of Iressa only in patients currently taking the drug and showing a response or via new clinical trials. The increased control required over distribution of the drug resulted in a near-complete drop in revenue generated in the US. With a much more limited patient population [EGFR mutations contribute to roughly 20% of NSCLC (Agarwal, 2012)], AstraZeneca forged ahead to change their commercialization strategy in countries outside the US. Interestingly, despite the clear link between EGFR mutation status and response, restrictions for use were not altered in Japan and Iressa was approved for use in China, mostly likely based on its efficacy in Asian populations. To expand to approval in Western countries, AstraZeneca positioned Iressa as both a second-line therapy to Taxotere (docetaxel) and partnered with the diagnostics company, DxS (which was subsequently bought by QIAGEN), to develop a molecular based assay for genotyping of EGFR. In 2009, Iressa was approved by the European Union with the CDx therascreen EGFR RGQ RT-PCR Kit. As of 2011, Iressa sales in Europe were ~$150 million, with a total of ~$520 million globally (Agarwal, 2012).

The “sea change” toward the utilization of CDx for FDA submission that resulted from the Iressa story, and others not discussed in this review, was ultimately driven by risk mitigation - mainly mitigation of the possibility of FDA rejecting approval of a therapeutic due to the lack of use of a CDx. FDA took a formal stance on CDx with the release of a draft guidance in 2011, which was subsequently finalized in August 2014 (http://www.fda.gov/downloads/MedicalDevices/DeviceRegulationandGuidance/GuidanceDocuments/UCM262327.pdf). This guidance defines an *In Vitro* CDx (IVD), indicates that in most instances a drug and the CDx should be FDA approved contemporaneously, outlines the FDA's regulatory enforcement policy and regulatory approval pathways for CDx, and discusses the implementation of labeling requirements upon co-approval of a drug and IVD assay. It goes without saying that the FDA oversight is not uniquely positioned to regulate only oncology therapeutics. Therefore, as part of each and every drug development program, from oncology to CNS, there could be questions about whether the drug can be shown to be safe and effective without enrichment of a particular patient population.

### Study outcomes as a result of personalized medicine approaches

There are significant financial benefits to utilizing a CDx strategy throughout drug development. Following the discovery of ALK-driven NSCLC in ~5–10% of the patient population (Soda et al., 2007), Pfizer proceeded with utilizing a fluorescence *in situ* hybridization (FISH) assay to detect the ALK chromosomal translocation as an eligibility requirement for patient enrollment. What would have taken years, hundreds millions of dollars, and thousands of patients to complete, took less than 3 years and less than 350 patients to achieve statistical power for regulatory submission and approval (Kwak et al., 2010). The short timeline and small patient enrollment is not unique to the ALK story. As shown in Table 2 multiple therapeutics have rapidly progressed from Phase I to market in less than 5 years through the use of patient enrichment with CDx.

**Table 2:** **Examples of rapidly-progressing therapeutics**.

**Company**	**Therapeutic**	**Phase I**	**Approval**
Roche/Genentech	Vemurafenib (Zelboraf™)	November 2006	August 2011
Pfizer	Crizotinib (Xalkori®)	December 2007	August 2011
GSK	Trametinib (Mekinist™)	May 2008	May 2013
Roche/Genentech	Ado-trastuzumab emtansine (Kadcyla®)	June 2009	February 2013
GSK	Dabrafenib (Tafinlar®)	April 2009	May 2013

Although there are less than 20 FDA Approved (Pre-Market Approval) CDx on the market to date (http://www.fda.gov/MedicalDevices/ProductsandMedicalProcedures/InVitroDiagnostics/ucm301431.htm), there are over 100 drugs with pharmacogenomics information within the label (Personalized Medicine Coalition, 2014) and a projected 30% of drugs in late clinical development rely on biomarkers for patient enrollment (http://www.personalizedmedicinecoalition.org/Userfiles/PMC-Corporate/file/pmc_personalized_medicine_by_the_numbers.pdf). One group has examined the effectiveness of utilizing a CDx during 676 clinical trials with 199 compounds. Utilizing a biomarker-driven approach to Phase III trials, the success rate increased from 28% (no biomarker) to 62% (Falconi et al., 2014; Olsen and Jorgensen, 2014). Therefore, utilizing a biomarker-driven clinical trial increased the success rate of Phase III trials, and subsequent approval, almost 2.5-fold.

### Reimbursement pressure

As discussed earlier, initially the narrowing of oncology patient populations appeared to translate to a loss in revenue potential for the therapeutic. However, with the evolution of regulatory agencies requiring patient enrichment to show safety and efficacy, and the increasing amount of data supporting faster and more successful routes to drug approval using CDx, the thinking has changed from assays being “burdensome” to assays being “useful” toward successful drug development. In addition, pressure is increasingly being put on pharmaceutical companies by insurance providers. Generally, targeted therapies are significantly more costly to a patient than generalized therapies; one report approximates the average cost per month for a branded oncology drug has doubled in the U.S. from $5000 to $10,000 in the past decade (http://www.imshealth.com/deployedfiles/imshealth/Global/Content/Corporate/IMS%20Health%20Institute/Reports/Secure/IMSH_Oncology_Trend_Report.pdf). In order to justify reimbursement of such expensive targeted therapies, insurance companies are requiring diagnostic tests be performed prior to prescription of a therapeutic. In European countries, a high level of scrutiny has been placed on newly developed therapeutics in comparison to existing treatments. For example, the UK National Institute for Health and Care Excellence (NICE) does not recommend the ALK inhibitor, Xalkori® for use in ALK-driven NSCLC, based on the ruling that the drug does not offer value for money.

In order to align with the fast-paced personalized medicine trend in healthcare and to ensure that each patient receives the most appropriate treatment for their disease, payers should support the use of CDx. Promotion of standardization of coverage and value-based reimbursements; reimbursement strategies that cover research-based innovations, such as Next Generation Sequencing; and flexibility in medical coding and billing facilitate the use of personalized medicine approaches to ensure the right patient receives the right treatment at the right time.

## Personalizing therapeutic approaches for circadian disorders

As discussed above, several genetic mutations have been identified that lead to a primary circadian disorder. Many more diseases have concomitant circadian abnormalities, which we refer to as secondary circadian disorders. For both types of disorders, phenotyping of the circadian disruption prior to initiation of therapy is critical to success—timing is everything when it comes to treatment of circadian dysfunction. In fact, measuring multiple rhythms within an individual is essential, as some disorders result from a misalignment of endogenous rhythms (Lewy et al., 2007). Traditional methods for monitoring the circadian phase and period in ambulatory humans include activity and body position monitoring via wearable devices (Bonmati-Carrion et al., 2015) and body temperature via wrist skin temperature (Kolodyazhniy et al., 2012). In a laboratory setting, multiple blood or saliva sampling to assess melatonin or other hormone patterns has been employed (Keijzer et al., 2014). In addition, the recent description of the rhythmic expression pattern of nearly half of the genome (Zhang et al., 2014) provides the opportunity to develop more and potentially better biomarkers for assessing the phase and period of individuals. Finally, mobile smartphones provide a unique and potentially highly effective method to collect robust data on circadian rhythms from an individual (Roenneberg, 2013). Thus, collecting robust data on a patient's circadian phenotype is both technically feasible and critical to the successful treatment of a circadian abnormality.

## Tasimelteon, a circadian success story

Approved on January 31st, 2014, HETLIOZ® (tasimelteon) is the first FDA approved treatment for adults with Non-24-H Sleep-Wake Disorder (Non-24), which is a rare circadian disorder occurring mostly in the totally blind. Vanda reports HETLIOZ® U.S. sales grew to $5.2 million in the first full quarter after launch. However, the road to approval was winding, with several failed clinical trials in other indications before the drug successfully demonstrated efficacy in Non-24.

Tasimelteon is a melatonin receptor MT1/2 agonist that was originally discovered by BMS (previously known as BMS-214,778) and licensed to Vanda Pharmaceuticals. Vanda opened an IND in 2004 for Shift Work Disorder, Jet Lag Disorder (due to eastward travel) and DSPD, however to date Vanda has not sought approval for any of these indications. Following on the success of another melatonin agonist, ramelteon (Rozerem®), which is approved in the US for treatment of insomnia, tasimelteon was tested in clinical trials for sleep disorders, including a Phase II trial on circadian rhythm sleep disorders and several phase III primary insomnia trials. However, the drug failed to show significant efficacy in these clinical trials. Additionally, the drug was also considered as a treatment for depression.

After several years of frustratingly weak results in other indications, Vanda demonstrated that tasimelteon produced phase shifts in healthy adults (Neubauer, 2015). Vanda then began Phase III clinical trials of the drug in a population with Non-24. There are ~1,300,000 blind people in the United States and ~10% of these individuals have no light perception. Without light input these totally blind individuals free run, drifting in and out of phase with the environment, impairing their ability to work, disrupting family life and impacting their overall health. Tasimelteon treatment entrained a higher number of totally blind to a 24-h cycle vs. placebo and the drug was approved in the US and available to appropriate patients via specialty pharmacy (Neubauer, 2015).

Vanda faced several significant challenges in the clinical trials testing the efficacy of tasimelteon for Non-24. The first task was setting the appropriate enrollment criteria and developing a screening protocol to capture the desired subjects. The number of enrollment failures in the SET trial was high; 391 subjects were screened; yet only 84 were enrolled in the randomized trial (Lockley et al., 2013). Vanda did not study subjects with a *tau* shorter than 24 h even though it is reasonable to expect that subjects with a short *tau* would eventually respond to treatment once the timing of dosing coincided with a sensitive phase in the circadian rhythm. Subjects with *tau* shorter than 24 h were excluded from the study because the dosing regime was fixed to administer the drug at 1 h prior to bedtime, based on concerns about drug-induced somnolence. Even though Vanda has not done a PRC for the drug, they were concerned that they would be unable to show efficacy in patients with a short *tau* if the drug was always to be taken at 1 h prior to bedtime.

Timing of treatment was not always aligned to the sensitive circadian phase of the treated subject's rhythm. The drug was always taken at 1 h prior to bedtime and although Vanda had tried to initiate dosing when subjects were in phase, 16% of subjects were out of phase when dosing was started. Subjects that were out of phase at the start of trial took a longer time on treatment to see efficacy, which contributed to the length of trial (Lockley et al., 2013). Circadian *tau* at was measured at 1 month and 7 months of treatment in the RESET trial (Lockley et al., 2013). RESET took almost 2 years to complete.

A final challenge in the tasimelteon trial was the use of entrainment as an outcome measure; an endpoint that is more correctly considered a proof of mechanism for the drug, not a proof that the drug is a treatment for the disorder. Only clinical benefit, defined by an improvement in how patients feel, function or survive, constitutes an acceptable primary endpoint for registration of a drug. An improvement in sleep did constitute a positive clinical outcome. The tasimelteon-treated group had significant improvement in the duration and timing of nighttime sleep and a significant decrease in daytime napping. Thus, tasimelteon is now available to provide treatment for blind patients suffering from Non-24.

## Final perspective

Circadian biologists have been collecting human subject circadian data in the research setting for decades, but few circadian drug treatment trials have been attempted. The general population has a growing familiarity with circadian biology in general and with their own unique rhythms, thanks to smart phone apps and wearable personal activity trackers. Nevertheless, drug developers have not yet linked the profound impact of circadian dysfunction on health to an influx of circadian drugs into pharma pipelines. The growing awareness of the link between disrupted circadian rhythms and obesity necessitates developing clinical trial strategies to effectively demonstrate the therapeutic benefit of drugs that alter circadian rhythms (chronotherapies). Better education of clinical researchers in the science of circadian biology is essential to developing enrollment criteria that will effectively capture the appropriate patient population. It should be apparent that circadian therapies must also be appropriately timed; treatment must be adjusted to be consistent with the patient's circadian phenotype, requiring a high degree of physician expertise and skill in interpreting circadian rhythms and possibly even daily input on scheduling dosing. Technological advances in remote data collection and the growing acceptance of sharing personal data will aid in personalizing circadian therapies to the appropriate patient. As data collection is improved, it may become obvious that not all patients will benefit from a specific therapy, as occurred in oncology. Biomarker strategies must be incorporated early in development to reduce screening failures and improve the potential to see efficacy, thus reducing the number of failed clinical trials. Biomarkers in this sense do not have to be genetic markers, but could be a clear circadian phenotype based on activity or other rhythm data.

Finally, circadian biologists have a deep appreciation of the negative impact on overall health of misaligned and disrupted rhythms, but simply demonstrating correction of the rhythm will not be sufficient for approval of a drug. Circadian drugs must also demonstrate that clinically relevant endpoints are improved by circadian adjustments.

### Conflict of interest statement

Barbara A. Tate is the acting CEO of Armada Therapeutics. The authors declare that the research was conducted in the absence of any commercial or financial relationships that could be construed as a potential conflict of interest.
